# Syndecans in hematopoietic cells and their niches

**DOI:** 10.1152/ajpcell.00326.2024

**Published:** 2024-06-24

**Authors:** Matthew W. Hagen, Nicollette J. Setiawan, Kelsey A. Woodruff, Christina M. Termini

**Affiliations:** ^1^Translational Science & Therapeutics Division, Fred Hutchinson Cancer Center, Seattle, Washington, United States; ^2^Human Biology Division, Fred Hutchinson Cancer Center, Seattle, Washington, United States; ^3^Department of Laboratory Medicine and Pathology, University of Washington School of Medicine, Seattle, Washington, United States

**Keywords:** bone marrow niche, hematopoiesis, leukemia, proteoglycan, syndecan

## Abstract

Heparan sulfate proteoglycans are a family of glycoproteins that modulate cell signaling by binding growth factors and changing their bioavailability. Syndecans are a specific family of transmembrane heparan sulfate proteoglycans that regulate cell adhesion, migration, and signaling. In this review, we will summarize emerging evidence for the functions of syndecans in the normal and malignant blood systems and their microenvironments. More specifically, we detail the known functions of syndecans within normal hematopoietic stem cells. Furthermore, we discuss the functions of syndecans in hematological malignancies, including myeloid malignancies, lymphomas, and bleeding disorders. As normal and malignant hematopoietic cells require cues from their microenvironments to function, we also summarize the roles of syndecans in cells of the stromal, endothelial, and osteolineage compartments. Syndecan biology is a rapidly evolving field; a comprehensive understanding of these molecules and their place in the hematopoietic system promises to improve our grasp on disease processes and better predict the efficacies of growth factor-targeting therapies.

## INTRODUCTION

**Figure 1.**
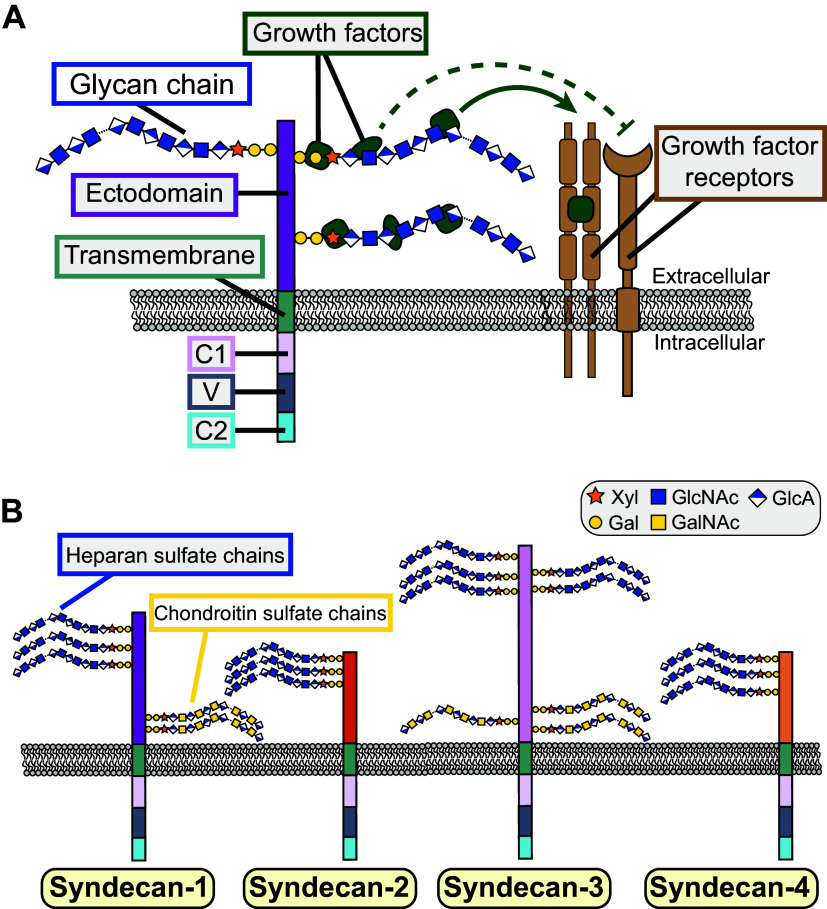
Syndecan structure and growth factor interactions. *A*: diagram of a syndecan core protein showing the ectodomain, transmembrane domain, variable V domain, and conserved C1 and C2 intracellular domains. Attached to the syndecan core protein are extracellular glycosaminoglycan chains, which bind growth factors. Growth factor binding to glycan chains promotes (arrow) or prevents (dotted line) growth factor binding to cognate growth factor receptors. *B*: the general structure of each syndecan family member is depicted. The potential sites for heparan sulfate or chondroitin sulfate glycan chains to be attached to each syndecan core protein are shown.

Our understanding of the structural and functional diversity of proteoglycans in mammalian organ systems has evolved dramatically since proteoglycans were first identified as a structural constituent of cartilage; this history has been eloquently reviewed elsewhere (1). Proteoglycans are a family of glycoproteins with a core protein decorated by distinct glycan chains. Syndecans are a specific proteoglycan class with structurally conserved transmembrane core proteins decorated by extracellular heparan sulfate and chondroitin sulfate moieties. Syndecan core proteins contain cytoplasmic and transmembrane features that bind other syndecans, the cytoskeleton, and protein/zonula occludens-1 (PDZ)-domain-bearing proteins. However, most of a syndecan molecule’s mass is derived from the extracellular glycan features that also define its action (2). The negatively charged environment created by syndecan-bound glycans restrains soluble growth factors and facilitates their interactions with their cognate cell-surface receptors (3) (Fig. 1*A*).

Despite the broad diversity exhibited by proteoglycans, the mammalian syndecan family contains just four structurally conserved members: syndecan-1, syndecan-2, syndecan-3, and syndecan-4 (Fig. 1*B*). Early reviews attempted to broadly categorize the syndecans by the tissues in which they are expressed, with syndecan-1 associated with early development, syndecan-2 with mesenchymal tissues, syndecan-3 with neural tissues, and syndecan-4 with broad expression across tissues (2). However, as described below, most syndecans are found across tissues and developmental stages. In terms of structure and sequence, syndecan-1 (CD138) and syndecan-3 can be considered one subfamily and syndecan-2 (CD362) and syndecan-4 another. Although all syndecans bear heparan sulfate glycan chains, only syndecan-1 and syndecan-3 can also bear chondroitin sulfate glycan chains. No global knockout mouse for any individual syndecan is lethal, but some exhibit moderate defects in development or recovery from injury (4).

Syndecans and other heparan sulfate proteoglycans (HSPGs) bear glycan chains with high structural variability. Glycan chain length, amount, and location of sulfation lend specificity to the interactions between HSPGs and growth factors (5, 6). The exostosin-like (EXTL) glycosyltransferases add an *N-*acetylglucosamine (GlcNAc) residue to an initiator polysaccharide to commence heparan sulfate (HS) chain formation. The EXTLs can also polymerize the glycan chains, but most of the polymerization is carried out by the EXT1/2 complex addition of alternating glucuronic acid (GlcA) and GlcNAc subunits. Following chain elongation, *N*-deacetylase-*N*-sulfotransferases (NDSTs) deacetylate and add sulfate groups to GlcNAcs. C5-epimerase converts many GlcA residues into iduronic acid. Finally, *O-*sulfate groups are added to the HS chain by sulfotransferases. There is one 2-*O* sulfotransferase (HS2ST1), three 6-*O* sulfotransferases (HS6ST1-3), and seven 3-*O* sulfotransferases (HS3ST1-7) that modify HS chains at the 2-, 6-, and 3-*O* positions, respectively (5).

The hematopoietic system depends upon growth factor signaling for homeostasis, regeneration, and protection from disease. At the top of the hematopoietic hierarchy is the hematopoietic stem cell (HSC), which is controlled through carefully coordinated HSC-intrinsic and HSC-extrinsic mechanisms (7). Through hematopoiesis, HSCs produce mature hematopoietic lineage cells; when hematopoiesis goes awry, hematological malignancies and disorders can develop. Healthy HSCs, mature hematopoietic cells, and malignant hematopoietic cells express syndecans (8). This review seeks to summarize recent findings related to syndecans’ cell-intrinsic functions. Hematopoiesis is also supported by the bone marrow microenvironment, or niche, which is a multicellular community that provides cues to hematopoietic cells to support their functions. Diverse cellular subtypes comprise the bone marrow niche, including mesenchymal stromal cells, endothelial cells, osteoblasts, osteoclasts, adipocytes, hematopoietic cells, and more. Syndecans are expressed across bone marrow niche cells and influence their function. This review will therefore also summarize recently defined cell-extrinsic functions of syndecans in the control of niche cell subtypes and support of hematopoietic cells.

## SYNDECANS IN HEALTHY HEMATOPOIETIC CELLS

### Hematopoietic Stem and Progenitor Cells

**Figure 2.**
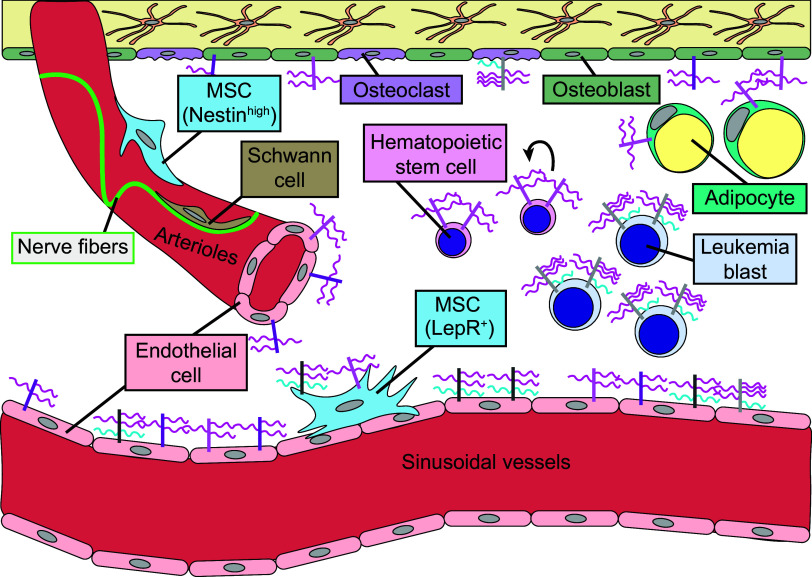
Model of syndecans in the bone marrow niche. The bone marrow niche consists of several cellular subtypes, including endothelial cells comprising sinusoidal blood vessels and arterioles. Nerve fibers and Schwann cells line arteriolar vessels. Perivascular stromal cells (Nestin^high^ or LepR^+^) are also present in the bone marrow, giving rise to adipocytes and bone cells. Osteoblasts (bone forming) and osteoclasts (bone resorbing) line the trabecular surfaces. Hematopoietic stem cells are a rare cellular subtype in the bone marrow. Leukemia blasts infiltrate the bone marrow, exhibiting aberrant signaling and uncontrolled growth. Bone marrow niche and hematopoietic cellular subtypes express syndecans.

Hematopoietic stem cells (HSCs) are rare self-renewing blood stem cells that give rise to our blood and immune systems throughout our lives. HSCs are supported by their niche, a microenvironment providing cellular and molecular cues to HSCs according to physiological demands (Fig. 2). Seminal work described an enrichment in *Sdc1* and *Sdc2* in the murine HSC compartment compared with more differentiated hematopoietic progenitors (7). More recently, it was shown that *Sdc2* was transcriptionally enriched in murine long-term HSCs. This enrichment was accompanied by a more than 10-fold increase in syndecan-2 surface expression on long-term HSCs compared with multipotent progenitors; syndecan-1, syndecan-3, and syndecan-4 were unchanged. Competitive transplantation assays revealed that syndecan-2+ HSCs had increased lineage repopulation ability and self-renewal capacity compared with HSCs that did not express syndecan-2. Mechanistically, knockdown analyses showed that syndecan-2 regulates HSC self-renewal via *Cdkn1c*-mediated control of HSC quiescence (9). Despite finding that syndecan-2 is enriched in the long-term HSC population, there is still much to discover about the role the syndecan family plays in hematopoietic regeneration. Understanding the role of syndecans may provide a novel target when considering how their function can be harnessed to improve bone marrow transplantation and regeneration for patients.

### Mature Hematopoietic Cells

The function and importance of surface proteoglycans do not wane once hematopoietic cells fully differentiate and enter systemic circulation. For example, there is growing evidence for associations between syndecan-1 and the identity and function of plasma cells, which was recently reviewed elsewhere (10). Furthermore, recent evidence demonstrates the need for a delicate balance of *Sdc1* expression in the T-cell arm of the lymphoid lineage. For example, murine *Sdc1* knockout T cells exhibited decreased proliferation in response to dendritic cell stimulation in an in vitro coculture study (11). Other reports indicated pathogenic roles for syndecan-1 in hematopoietic diseases; syndecan-1 surface expression was predictive of T-cell autoactivation in a murine lupus model (12). In addition, syndecan-1-expressing T cells promote disease progression by supporting autoreactive B-cell responses (13). These findings highlight recently acquired knowledge about the functions of syndecans in the lymphoid lineages. There is still much to be discovered about how syndecans impact normal and diseased myeloid cells and specialized subsets of mature hematopoietic cells.

## SYNDECANS IN DISEASED BLOOD CELLS

### Acute Myeloid Leukemia

Acute myeloid leukemia (AML) is a heterogeneous blood cancer that is characterized by an accumulation of primitive myeloid cells, termed blasts. AML is the most common acute leukemia in adults and is highly relapse-prone and chemotherapy-resistant, highlighting the need for a deeper understanding of AML biology to develop targeted therapies (14). Circulating syndecan-1 in AML is associated with signs of disease progression including bleeding, impaired platelet function, and leukocytosis. These risks contribute to endothelial perturbation and dysregulation of the endothelial glycocalyx (15). A recent clinical study found increased plasma syndecan-1 in patients with AML compared with healthy controls; however, it did not find differences in circulating syndecan-1 between patients achieving remission and those failing to achieve remission following chemotherapy. Furthermore, it showed no significant correlations between circulating syndecan-1 levels and complete blood counts (16). Additional targeted studies are needed to determine the exact roles of syndean-1 in AML cell function and disease progression.

Misexpression of heparan sulfotransferases modifying syndecans and other HSPGs is also associated with myeloid malignancies. The gene encoding the enzyme catalyzing 2-*O* heparan sulfation, *HS2ST1*, was found to be overexpressed in the SKM-1 myelomonocytic leukemia cell line. *HS2ST1* expression was dramatically decreased upon SKM-1 differentiation into granulocytes induced by all trans retinoic acid (17). This indicates that 2-*O* heparan sulfation may maintain SKM-1 cells in a primitive state and serve as a potential therapeutic target for myelomonocytic leukemia. Foundational studies also identified *HS3ST3B1*, which encodes an enzyme catalyzing 3-*O* sulfation, as promoting AML progression through induction of vascular endothelial growth factor (VEGF) expression and secretion (18). Further work is needed to determine whether these effects are mediated through sulfotransferase activity on syndecans or other HSPGs. Future work should focus on elucidating these intricacies to identify novel biomarkers and uncover a new level of cell signaling regulation to target therapeutically.

### Chronic Myeloid Leukemia

Like AML, primitive myeloid cell accumulation is also characteristic of chronic myeloid leukemia (CML). CML is distinct from AML because it develops more slowly and does not completely stop the development of mature blood cells, often resulting in less severe disease and more favorable outcomes for patients. Despite these favorable outcomes, CML stem cells cause disease relapse (19). New markers and therapeutics targeting resistant leukemia cell populations are necessary to prevent disease relapse and improve patient outcomes. Recent work identified syndecan-1 as an important marker for aggressive CML cell populations. Spinler and colleagues showed that antibody blocking of syndecan-1 on primary mouse CML cells significantly reduced leukemic colony formation. They then isolated wild-type (WT) or *Sdc1*-knockout murine HSCs and transduced them with a BCR-ABL/NUP98-HOXA9 CML oncogenic driver gene, finding that CML cells lacking *Sdc1* showed a similar reduction in colony formation. In addition, *Sdc1*-null murine CML cells engrafted less efficiently than control cells. Mice injected with *Sdc1*-null CML cells survived longer than mice injected with control CML cells (19). Mechanistically, *Sdc1* was shown to mediate integrin-dependent CML cell growth and migration. These data indicate that syndecan-1 may be a viable biomarker for disease risk or a potential clinical target to control CML. More work is needed to establish whether and how the entire proteoglycan repertoire influences leukemia cell functions and patient outcomes.

### Multiple Myeloma

Heparan sulfate proteoglycans also have key functions in nonleukemia hematological malignancies and bone marrow disorders. For example, *SDC1* has long been established to be prominently expressed by multiple myeloma plasma cells (20). However, recent work demonstrated that multiple myeloma cells expressing junctional adhesion molecule C exhibit low surface expression of syndecan-1 in murine bone marrow, challenging the notion that all multiple myeloma cells express syndecan-1 uniformly (21). Separate findings indicated that primary bone marrow stromal cells from patients with myeloma and the bone marrow stromal cell line HS5 express high levels of syndecan-1 and heparan sulfate (22). Furthermore, *EXT1* knockout in HS5 stromal cells reduces their CXCL12γ surface presentation. Functionally, it was determined that stromal cells present CXCL12γ via heparan sulfates, which, in cocultures, promotes multiple myeloma resistance to bortezomib, a current myeloma therapy. Although it is unclear whether increased heparan sulfation on syndecan-1 specifically mediates this effect, these findings provide significant insight into the multicellular roles of HSPGs and their modifications in regulating myeloma. Additional work should focus on elucidating the function of specific proteoglycans in myeloma cells and cells of their microenvironment in regulating disease pathogenesis and drug resistance.

### Lymphoma

An intriguing new concept for proteoglycan regulation of malignant hematopoiesis arose from a recent report exploring the interactions between B-cell acute lymphoblastic leukemia (B-ALL) and stromal cells (23). In this report, Karantanou and colleagues interrogated the function of the endolysosomal adapter, pleckstrin homology domain family M member 1 (*Plekhm1*), in the bone marrow microenvironment in regulating B-ALL. They used a mouse model to induce BCR-ABL1+ B-ALL, which was transplanted into wild-type or *Plekhm1* knockout mice. They identified significantly worse disease progression when B-ALL cells occupied the *Plekhm1* knockout microenvironment, which occurs via altered small extracellular vesicle (sEV) content from *Plekhm1i* mesenchymal stromal cells. These researchers investigated the contents of sEVs, finding a robust increase in syndecan-1 within the sEVs. This exciting report indicates that the transfer of proteoglycans may be a mechanism by which malignant hematopoietic cells can hijack signaling processes to propagate disease more effectively.

### Bleeding Disorders

Recent work demonstrated that patients with immune-mediated thrombotic thrombocytopenic purpura had significantly increased expression of plasma syndecan-1 compared with healthy controls (24). This work determined that increased plasma syndecan-1 expression was associated with increased mortality. Although these clinical cohort studies identified syndecan-1 as a biomarker or predictor of patient outcomes, the function of syndecan-1 in thrombotic thrombocytopenic purpura remains undefined. Future studies should work to elucidate the function of syndecans in hemostasis, focusing on the mechanistic interplay between syndecans expressed by blood cells and the vasculature.

## SYNDECANS IN HEMATOPOIETIC NICHE CELLS

### Mesenchymal Stromal Cells

Bone marrow mesenchymal stromal cells (MSCs) give rise to osteoblasts, chondrocytes, and adipocytes. Bone marrow MSCs support HSCs to maintain their self-renewal and regenerative capacity (25). MSC-intrinsic roles for syndecans in MSC functions have recently been discovered. For example, *SDC1* knockdown in primary human MSCs skewed their differentiation toward an adipocyte-forming phenotype (26). Furthermore, *SDC1* knockdown in actively differentiating MSCs similarly resulted in increased expression of *Adipoq*, an adipogenic marker. This suggests that syndecan-1 regulates the sensitive balance between osteoblast and adipocyte differentiation from MSCs, which is relevant in systemic metabolic disorders (27). Further elucidating the mechanism by which *SDC1*, and possibly other syndecans, regulates differentiation of MSCs could lead to new therapeutic targets in metabolic disorders such as obesity.

Syndecan-2 is more highly expressed than other syndecan family members in cultured bone marrow-derived human MSCs (28). In a study examining the potential of these cells for cell therapy, the authors intravenously delivered them to septic mice, promoting mouse survival and bacterial clearance. They tested the function of MSC *SDC2* in the process of bacterial clearance by transplanting control and *SDC2* knockdown MSCs generated with lentiviral approaches. They determined that the loss of *SDC2* decreases inflammatory resolution, bacterial clearance, and neutrophil phagocytosis, concluding that *SDC2* is critical for the cellular and paracrine functions of therapeutically delivered MSCs during sepsis; the mechanisms by which this occurs remain undefined (28). Conversely, *Sdc3* expressed by murine BM MSCs supports proinflammatory effects in joints. Bone marrow-derived MSCs were compared between wild-type and *Sdc3*-null mice, revealing enhanced collagen type-I adhesion by the *Sdc3*-lacking MSCs relative to wild types. MSC injection into the joints of mice with antigen-induced inflammatory arthritis revealed that *Sdc3*-null MSCs had increased capacity to repair cartilage compared with wild-type cells (29). The authors postulated that this occurs via altered AKT and ERK signaling potency. Further work should aim to detail the mechanisms allowing syndecans to promote or attenuate inflammatory responses toward identification of potential therapeutic targets.

Although syndecans found on bone marrow MSCs are linked to several diseases such as diabetes, arthritis, and disorders of bone density, their function in hematopoietic homeostasis and regeneration is not well defined. Since HSPCs interact with and are influenced by signals from their microenvironment, elucidating the role of syndecans on stromal cells could provide new avenues to improve HSC maintenance, expansion, or regeneration after injury.

### Endothelial Cells

Vascular endothelial cells form the functional interface between flowing blood and static tissue throughout mammalian organ systems, including the bone marrow. Key to the boundary function of the endothelium is the glycocalyx, a luminal collection of proteoglycans and other macromolecules. The glycocalyx defines the electrostatic gradients in the boundary regions of all blood vessels, broadly influencing the ability of circulating molecules and cells to interact with the endothelium and its underlying tissues (30). Elements of the glycocalyx also act as mechanosensors and facilitators of receptor-ligand interactions. Syndecans are known to regulate growth and permeability across vascular biology (31).

Perhaps the most well-characterized endothelial cell signaling pathways involve vascular endothelial growth factors (VEGFs) and their cognate tyrosine receptor kinases (VEGFRs), which modulate angiogenesis and vascular permeability (32). Syndecan-2 participates heavily in normal angiogenesis and arteriogenesis during development and in response to injury. Six-day-old mice lacking endothelial *Sdc2* displayed reduced retinal endothelial progression relative to controls (33). The authors assessed recovery from hindlimb ischemia in adult mice, finding reduced arteriogenesis and weaker perfusion after recovery in endothelial *Sdc2* knockout mice compared with controls. Neither early vascular growth nor adult injury recovery was affected in global *Sdc4* null mice, which the authors attributed to the greater degree of 6-*O*-heparan sulfation on the syndecan-2 heparan sulfate chains. Knockout of *Sdc-2* or *Sdc-4* also reduced injury responses and increased vascular permeability. *Sdc2* knockout mice exhibited reduced Evan’s blue extravasation upon bolus injection with certain VEGF isoforms and increased neuroprotection in experimentally induced stroke compared with control mice (34). Another group demonstrated that *Sdc4*, which is positioned near endothelial junctions, is uniquely upregulated during malignant angiogenesis (35). Furthermore, *Sdc2* deletion from endothelial cells reduces the internalization of VE-cadherin, which is critical for enhanced permeability and early angiogenesis in this maladaptive process. Considering these important revelations about VEGF-syndecan interactions across the endothelium, targeted work examining the unique roles of these pathways in the hematopoietic niche microvasculature is called for.

Finally, the glycocalyx contributes to endothelial cell mechanical sensitivity. *Sdc4* knockdown in cultured human umbilical vein endothelial cells prevents cells from aligning parallel to applied unidirectional fluid shear stress. Meanwhile, *Sdc4* global knockout in mice similarly inhibited alignment of their aortic endothelium and increased their atherosclerotic plaque burden (36). The relationship between syndecan-4 and mechanical stress may explain the increase in soluble plasma syndecan-4 observed in patients with treatment-resistant hypertension compared with healthy controls (37). A more recent characterization of molecular tension in syndecan-1 triggered by mechanical cues and fluid shear stress demonstrates that multiple syndecans can function as endothelial mechanosensors (38). Still unclear is the importance of these mechanosensitive systems in organ system microvasculature.

### Osteoblasts/Osteoclasts

Bone marrow stromal cells can differentiate into osteoblasts, which build new bone during development or healing. Osteoblasts are vital in maintaining bone architecture throughout the mammalian lifespan (39). It was observed that adult global *Sdc3*-null mice had low bone volume, reduced bone formation, and increased bone fragility compared with wild types (40). This phenotype was found to be a consequence of delayed osteoblast maturation and impaired osteoblast function via diminished canonical WNT signaling. As this work relied upon a global knockout mouse, it is unclear how the cellular source of syndecan-3 impacts this regulatory pathway. To maintain normal bone structure and remodeling, osteoblasts interact with osteoclasts, a subset of cells responsible for dissolving old or damaged bone, to facilitate a cycle of degradation of old bone tissue and the formation of new bone. *Sdc1* global knockout mice had similar calvarial osteoblast and osteoclast frequencies. However, upon coculture of primary mouse osteoblast and osteoclast precursors in differentiation media, *Sdc1* knockout in osteoblast precursors reduced osteoblastogenesis. They also found that syndecan isoforms in the *Sdc1* knockout osteoblasts were expressed at comparable levels compared with WT osteoblasts. The authors postulated that this occurs via soluble ligand availability via receptor activator of nuclear factor-kappaB ligand and osteoprotegerin (41). Although syndecan-1 and syndecan-3 have been characterized in osteoblast and osteoclast maintenance, we still have limited knowledge about their influence on other cells in trabecular and bone marrow environments, such as hematopoietic or stromal cells. A better understanding of the syndecan family and its role in bone formation and regeneration could unveil therapeutically targetable mechanisms relevant to diseases, including bone cancers and osteoporosis.

### Syndecans in Other Niches

The role of syndecans in signal regulation is not limited to the hematopoietic system and extends beyond blood stem cell niches (42). Significant work has focused on the interplay between central nervous system mesenchymal stromal cells, neurogenesis fate decisions, and HSPGs (43, 44), particularly syndecan-1 (45), whereas syndecans 3 and 4 have been found to mark the satellite cells that enable new growth in adult skeletal muscle (46). Unclear is whether these other contributions point toward a conserved role for syndecan-mediated signaling across adult stem cell functions.

## CONCLUSIONS

In recent years, the field of syndecan biology has appropriately focused its energy on the roles of these complex proteoglycans as critical facilitators and regulators of signaling between soluble ligands and their cognate cell-surface growth receptors across organ systems. Much of this work has used genetic knockout models both in vivo and in cultured cells, with a growing emphasis on the use of tissue-specific conditional knockouts. As the field evolves, our attention should turn to the roles of syndecans as regulators of intercellular signaling, especially in tightly regulated cellularly diverse environments such as the hematopoietic bone marrow niche.

## GRANTS

This project was supported by the National Institutes of Diabetes and Digestive and Kidney Diseases (5K01DK126989 to C.M.T.), the Andy Hill Cancer Research Endowment Fund (FY23-DR-01), and the American Heart Association (23CDA1039196).

## DISCLOSURES

No conflicts of interest, financial or otherwise, are declared by the authors.

## AUTHOR CONTRIBUTIONS

C.M.T. prepared figures; M.W.H., N.J.S., K.A.W., and C.M.T. drafted manuscript; M.W.H., N.J.S., K.A.W., and C.M.T. edited and revised manuscript; M.W.H., N.J.S., K.A.W., and C.M.T. approved final version of manuscript.
